# Molecular Dissection of Crz1 and Its Dynamic Subcellular Localization in *Cryptococcus neoformans*

**DOI:** 10.3390/jof9020252

**Published:** 2023-02-14

**Authors:** Benjamin J. Chadwick, Brittain Elizabeth Ross, Xiaorong Lin

**Affiliations:** 1 Department of Plant Biology, University of Georgia, Athens, GA 30602, USA; 2 Department of Microbiology, University of Georgia, Athens, GA 30602, USA

**Keywords:** *Cryptococcus neoformans*, calcineurin, Crz1, stress granules, intrinsically disordered regions

## Abstract

Across lower eukaryotes, the transcription factor Crz1 is dephosphorylated by calcineurin, which facilitates Crz1 translocation to the nucleus to regulate gene expression. In the fungal pathogen *Cryptococcus neoformans*, calcineurin–Crz1 signaling maintains calcium homeostasis, thermotolerance, cell wall integrity, and morphogenesis. How Crz1 distinguishes different stressors and differentially regulates cellular responses is poorly understood. Through monitoring Crz1 subcellular localization over time, we found that Crz1 transiently localizes to granules after exposure to high temperature or calcium. These granules also host the phosphatase calcineurin and Pub1, a ribonucleoprotein stress granule marker, suggesting a role of stress granules in modulating calcineurin–Crz1 signaling. Additionally, we constructed and analyzed an array of Crz1 truncation mutants. We identified the intrinsically disordered regions in Crz1 contribute to proper stress granule localization, nuclear localization, and function. Our results provide the groundwork for further determination of the mechanisms behind the complex regulation of Crz1.

## 1. Introduction

Crz1 is an important downstream transcription factor in calcineurin signaling in lower eukaryotes [1]. Deletion of the *CRZ1* gene in pathogenic fungi has pleiotropic effects, with increased sensitivity to calcium, cell wall stress, and attenuated virulence being common phenotypes. This has been observed in the rice blast fungus *Magnaporthe oryzae* [2], in the opportunistic human fungal pathogens *Aspergillus fumigatus* [3,4], *Candida* species [5,6], and *Cryptococcus neoformans* [7,8,9,10]. In *C. neoformans*, the calcineurin–Crz1 pathway is also critical for the yeast to hypha transition induced by glucosamine [11].

Crz1 translocates from the cytosol to the nucleus after calcineurin-dependent dephosphorylation in the model yeast *Saccharomyces cerevisiae* [12]. This phosphorylation-dependent regulation of Crz1′s subcellular localization appears conserved across lower eukaryotes. In *C. neoformans*, Crz1 may translocate to the nucleus in response to 37 °C, glucosamine, or calcium stress. In addition to trafficking between the cytosol and the nucleus, a punctate localization of Crz1 has been observed in response to high salt or high temperature shock [8,11]. How the subcellular localization of Crz1 and its function are differentially regulated in response to different stresses is poorly understood. *C. neoformans* Crz1 protein is ~1100 amino acids in length with a C-terminal DNA binding domain ~90 amino acid long. The remaining ~1000 amino acids of the protein are mostly predicted to exist in an unfolded or disordered state. Disordered protein sequences have been shown to contribute to phase separation as well as transcription factor DNA binding activity [13,14,15,16]. It was recently demonstrated in the fungal pathogen *Candida albicans*, that the intrinsically disordered regions of the phosphatase Ptc2 allow it to phase separate in response to host CO_2_ levels [17]. Because Crz1 contains many regions of intrinsic disorder with unknown function, we decided to investigate the molecular basis of Crz1 function and its subcellular localization by creating and examining Crz1 mutant proteins with truncations of different regions. Here, we report our systematic characterization of these mutant Crz1 proteins’ localization in response to calcium, glucosamine, heat, and salt stress, and their ability to complement the phenotypes of the *crz1*Δ mutant.

## 2. Materials and Methods

### 2.1. Strains and Media

All *C. neoformans* strains used are listed in Supplemental Table S1. Cryptococcal cells were freshly thawed from 15% glycerol stocks stored at −80 °C and cultured on YPD medium (1% yeast extract, 2% BactoPeptone, and 2% dextrose) unless specified otherwise. For all growth assays involving comparisons of different strains, the cells were first adjusted to the same optical density (OD) by quantification of the OD_600_ with a spectrophotometer or with a Biotek Epoch 2 plate reader. For the spotting assays testing growth in hypoxic conditions, an environment of 37 °C, 0.1% O_2_, and 5% CO_2_ was maintained with a Biospherix C chamber with a Pro-Ox controller and a Pro-CO_2_ controller to maintain O_2_/CO_2_ levels (Biospherix, Lacona, NY, USA).

### 2.2. Genetic Manipulation

The plasmids and primers used in this study are listed in Supplemental Table S1. To overexpress and fluorescently label Crz1, the open reading frame of *CRZ1* was first PCR amplified and cloned into the vectors with mCherry and mNeonGreen for tagging after restriction enzyme digestion. The vectors contain the *GPD1* promoter for constitutive overexpression and a neomycin (NEO) drug selection marker. Internally truncated *CRZ1* mutant alleles were generated by overlap PCR (or fusion PCR). The primers used for each construct are listed in Supplemental Table S1. All mutant *CRZ1* allele constructs and the wild-type allele construct were cloned into the same plasmid backbone, pUC19, with expression controlled by the *GPD1* promoter and with a C-terminal mNeonGreen tag. The constructs were introduced into the *C. neoformans crz1*Δ mutant genome by TRACE (transient CRISPR-Cas9 coupled with electroporation) [18,19] and transformants were selected on 100 μg/mL of G418. All constructs in the selected transformants were integrated into the “safe haven” locus *SH2* [19,20].

### 2.3. Microscopy

*C. neoformans* strains were observed under a Zeiss Imager M2 microscope, and all images were acquired by an AxioCam MRm camera and processed with Zen pro software version 3.1 (Carl Zeiss Microscopy, Jena, Germany). For heat shock experiments, the cells grown overnight at 22 °C were prepared for microscopy on a 42 °C pre-heated glass slide and incubated at 42 °C for 15–20 min. For the high calcium and salt shock experiments, the cells were suspended in water containing 1 M NaCl or 100 mM CaCl_2_ for 15–20 min. The cells were then immediately examined under the microscope. For timelapse experiments involving heating, the cells were prepared on a pre-warmed slide and examined on the same Zeiss Imager M2 microscope equipped with a heated stage (PE120 Linkam stage, McCrone Microscopes & Accessories). For timelapse experiments testing responses to CaCl_2_, the cells were suspended in 1 mM CaCl_2_ on a microscope slide and immediately imaged.

## 3. Results

### 3.1. The crz1Δ Mutant Displays Pleiotropic Growth Defects

**Figure 1:**
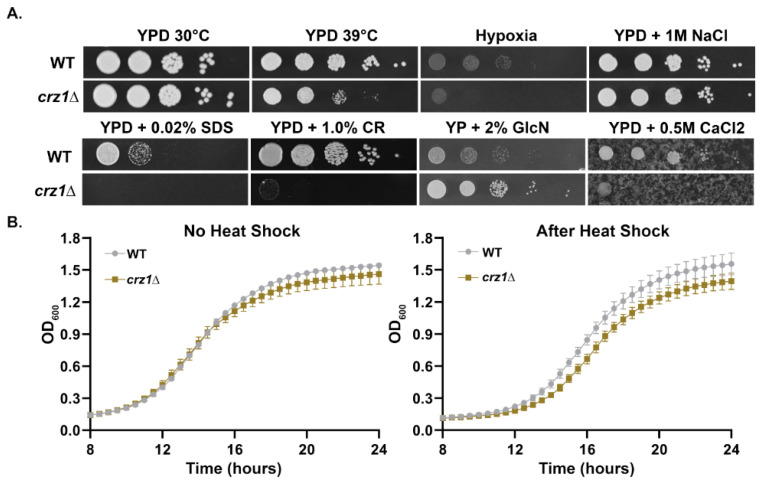
Growth assays of the *crz1*Δ mutant under various stress conditions. (**A**) The WT strain H99 and the *crz1*Δ mutant were grown overnight in liquid YPD at 30 °C, washed, adjusted to the same cell concentration, serially diluted, and spotted onto the media and incubated under the indicated conditions. Images were taken two days after incubation. (**B**) The overnight cultures of H99 and the *crz1*Δ mutant were split into two groups and diluted to OD_600_ = 0.1 in YPD medium. Three replicates of each strain were included per group. One group was inoculated into a 24-well microplate and incubated at 30 °C with double orbital shaking in a Biotek Epoch 2plate reader. Growth was monitored every 30 min by measuring OD_600_. The second group was heat shocked by incubation at 42 °C for 20 min in a thermocycler prior to monitoring growth at 30 °C in the Epoch 2.

We first tested the growth of the *crz1*Δ mutant in various stressful conditions. The *crz1*Δ mutant grew poorly at 39 °C and under hypoxic conditions (Figure 1A). In addition, the *crz1*Δ mutant was sensitive to SDS (membrane disrupting detergent), Congo Red (cell wall stressor), and high calcium (Figure 1A). Our observation is consistent with previous findings that deletion of the *CRZ1* gene in *C. neoformans* led to increased sensitivity to cell wall stresses, high temperature, calcium, and hypoxia [7,8,10,21]. However, the *crz1*Δ mutant showed no growth defects in 1 M NaCl and it grew noticeably better than the WT strain on the filamentation-inducing media YP + Glucosamine. This is likely due to the fact that the *crz1*Δ mutant grows in the yeast form on this medium while H99 can grow filamentously and the filamentous growth rate is slower relative to yeast growth rate in *C. neoformans* [11].

As previously described, the *crz1*Δ mutant grew similarly as the wild type at 30 °C but poorly at 39 °C after two days of incubation on plates at the constant temperature, indicating that Crz1 is important for thermotolerance. To test if Crz1 is important for adaptation after a brief heat shock, we tested the recovery growth of both WT H99 and the *crz1*Δ mutant at 30 °C after 20 min of incubation at 42 °C in liquid culture. When cultured at the constant temperature of 30 °C without heat shock, there was a slight growth defect of the *crz1*Δ mutant compared to the WT strain (left graph, Figure 1B). Although the WT grew to a slightly higher optical density, the time spent in lag phase growth was about the same (both ~10 h). However, with the short heat shock at 42 °C, the growth defect of the *crz1*Δ mutant was exacerbated (Figure 1B). The *crz1*Δ mutant experienced a lag growth phase about 30 min longer than the WT (*p* < 0.0001, two-tailed test). These results indicate that Crz1 is important for heat shock adaptation even after a short exposure to high temperature.

### 3.2. Crz1 Co-Localizes to Stress Granules with Pub1 and Calcineurin in Response to Heat or Salt Stress

**Figure 2:**
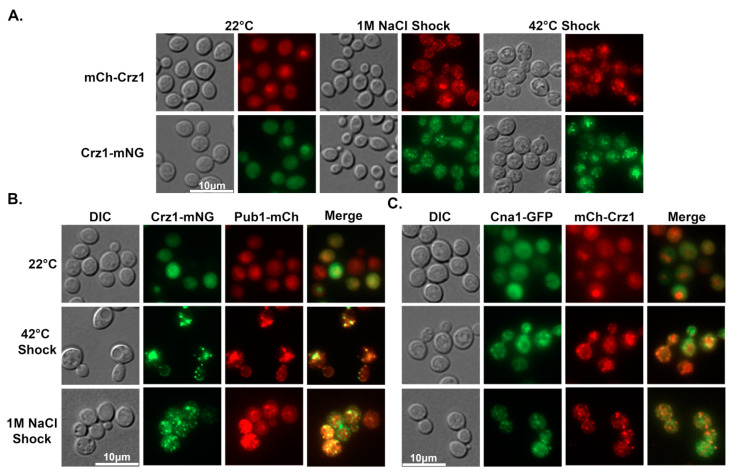
Crz1 co-localizes to stress granules with Pub1 and calcineurin in response to heat or salt shock. (**A**) mCherry-Crz1- or Crz1-mNeonGreen-expressing cells were incubated at 22 °C overnight and fluorescence was observed. To test the effects of salt and heat shock, cells were suspended in 1 M NaCl or exposed to 42 °C for 15–20 min prior to microscopic examination. (**B**) The same procedure was done with cells expressing both Crz1-mNeonGreen and Pub1-mCherry and (**C**) cells expressing both mCherry-Crz1 and Cna1-GFP. The scale of all images in each panel is the same.

Under non-stimulating conditions, Crz1 showed diffused cytoplasmic localization in most cells and enriched nuclear localization in a small proportion of the population (Figure 2A), as we expected based on our previous study [11]. In response to heat (42 °C) or salt (1 M NaCl) shock, we found that both an N-terminal mCherry-tagged Crz1 fusion protein (mCh-Crz1) and a C-terminal mNeonGreen-tagged Crz1 fusion protein (Crz1-mNG) localized to granules in all cells (Figure 2A). Previously, Kozubowski et al. found that calcineurin (Cna1) co-localizes with the polyA-binding protein Pub1 in stress granules at 37 °C [22]. Pub1 is a known marker for stress granules in response to starvation, heat shock, or acidification [23]. Because Crz1 is a known downstream target of calcineurin, we decided to test if Crz1 co-localizes with calcineurin catalytic subunit Cna1 or Pub1 at high temperatures or in high salt. To that end, we introduced the Crz1-mNG into a strain harboring Pub1-mCherry and the mCh-Crz1 into a strain harboring Cna1-GFP. Under the non-stimulating control condition (22 °C), Crz1 and Pub1 were mostly diffused in the cytoplasm with some cells showing enrichment in the nucleus (Figure 2B, top left images). By contrast, calcineurin Cna1 was in the cytoplasm and likely excluded from the nucleus (Figure 2C, top right images). In response to 42 °C or salt shock, we found that Crz1 localized to granules and these granules co-localized with Pub1 (Figure 2B) and calcineurin Cna1 (Figure 2C). Taking these observations into consideration, we hypothesize that co-localization of Crz1 and the phosphatase calcineurin to stress granules may be a mechanism to promote their interaction and facilitate dephosphorylation of Crz1 and its subsequent translocation to the nucleus.

### 3.3. Crz1 Localizes to Stress Granules at Host Physiological Conditions

**Figure 3:**
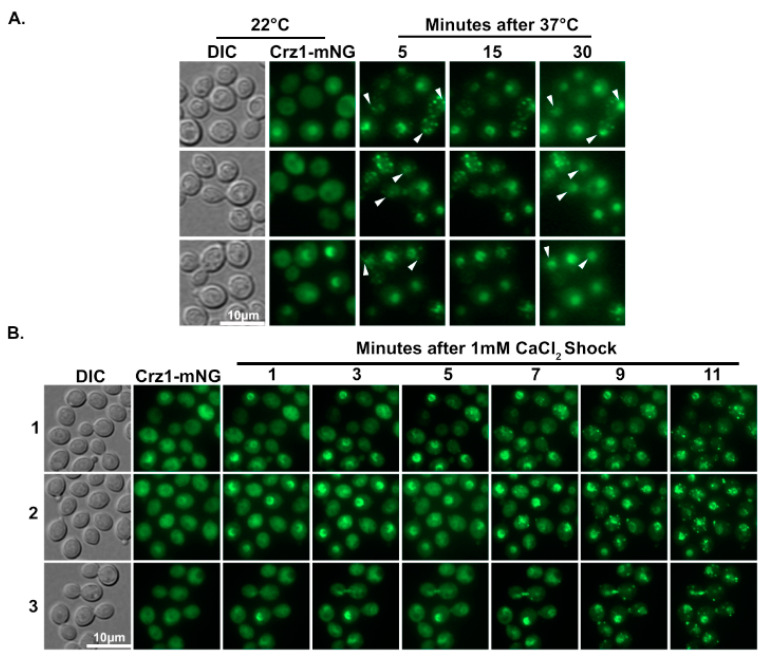
Crz1 localizes to granules at conditions relevant to host physiology. (**A**) Cells expressing Crz1-mNeonGreen were initially grown overnight at 22 °C. The cells were then prepared for microscopy on a 37 °C pre-heated glass slide and examined on a microscope equipped with a heated stage set to 37 °C. White arrowheads indicate examples of cells where Crz1 localized to granules first before typical nuclear localization in response to 37 °C. (**B**) The same cells grown overnight at 22 °C were suspended in 1 mM CaCl_2_ on a microscope slide and immediately imaged (Crz1-mNG). Images were taken of the same field of view at the indicated times. The scale is the same for all images in the same panel.

Crz1 was previously found to localize to the nucleus when cells were grown overnight at the mammalian host temperature of 37 °C or after being exposed to 100 mM CaCl_2_ [8,11]. Here we found that Crz1 localizes to stress granules after a short exposure to high temperature and salt. We wondered if the seemingly conflicting localizations of Crz1 are due to dynamic trafficking of Crz1 and the differences in the timing of observation in previous studies and this study. We therefore monitored Crz1-mNeonGreen localization in the same cells over time in response to a temperature shift from 22 °C to 37 °C. Interestingly, after 5 min of 37 °C heat shock, Crz1 was found in puncta in some cells (Figure 3A). The Crz1 in the same cells then later localized to the nucleus after 30 min (arrow heads in Figure 3A). This result further supports our hypothesis that stress granule localization facilitates Crz1 translocation to the nucleus, likely by promoting interaction between Crz1 and calcineurin. This punctate-to-nucleus translocation was not observed when cells were exposed to 100 mM CaCl_2_. Because *Cryptococcus* is likely exposed to much lower concentrations of calcium in the host than the 100 mM CaCl_2_ used in earlier studies (e.g., CaCl_2_ concentration in serum is ~2.2–2.6 mM), we then tested Crz1 localization in response to 1 mM CaCl_2_. We noticed that ~33% of cells displayed nuclear localized Crz1 in less than 1 min of exposure to exogenous calcium at 1 mM, and ~80% of cells displayed punctate Crz1 localization after 5 min (Figure 3B, Supplemental Videos S1 and S2). We monitored Crz1 localization in response to 1 mM CaCl_2_ for up to 30 min and found the puncta localization was stable for the whole duration. We observed similar puncta localization of Crz1 after exposure to 10 mM CaCl_2_. Thus, it seems that granular localization of Crz1 likely occurs in the host.

### 3.4. Crz1 Localizes to the Mother–Daughter Bud Neck in Response to Stress Conditions

**Figure 4:**
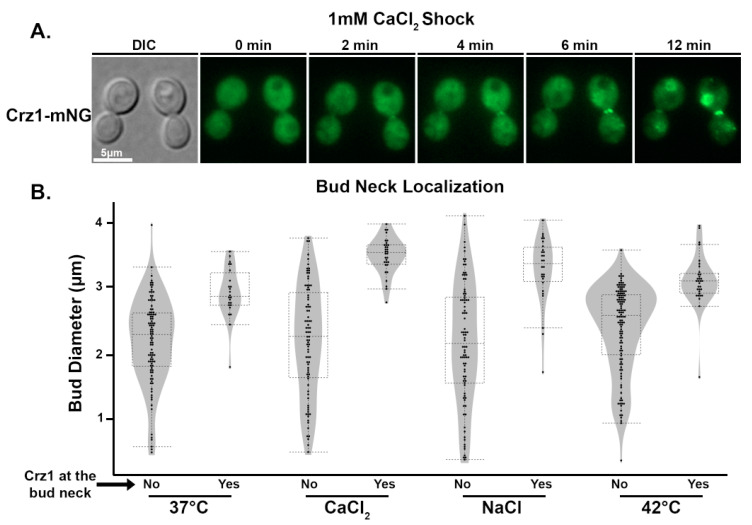
Crz1 localizes to the bud neck in response to stress. (**A**) Cells expressing Crz1-mNeonGreen were suspended in 1 mM CaCl_2_ on a microscope slide and immediately imaged. The same field of view was captured at the indicated times and representative images are shown. (**B**) Cells with buds were characterized as having Crz1-mNG localization at the bud neck or not. The diameter of each bud was measured using ZEN 3.1 software. Over 100 cells were quantified per condition tested.

While subjecting cryptococcal cells to a variety of stresses, we noticed localization of Crz1-mNeonGreen to the bud neck of replicating cells (mother cells with daughter buds). At room temperature without any added stress, Crz1 localization to the bud neck was not observed. To examine the dynamics of this bud neck localization, we monitored Crz1-mNeonGreen over time after exposure to a 1 mM CaCl_2_ shock (Figure 4A, Supplemental Video S3). For a period of 12 min, gradual accumulation of Crz1-mNeonGreen signal in both the nucleus and the bud neck was observed. We further tested bud neck localization of Crz1 in response to 37 °C, 1 M NaCl, 1 mM CaCl_2_, and 42 °C heat shock. For each condition, we analyzed over 100 mother–daughter pairs and categorized them as showing Crz1 bud neck localization or not. We further measured the diameter of the budding daughter cells to test if Crz1′s bud neck localization correlates with the size of buds. Interestingly, Crz1′s bud neck localization was observed in response to each stress tested, and it was primarily observed in a subset of the cells which have larger buds (Figure 4B). The median bud length measured for all conditions was ~2.6 μm, with the minimum and maximum lengths found being 0.5 μm and 4.0 μm, respectively. Over 20% of all budding cells and over 60% of cells with a diameter over 3 μm displayed Crz1 localization at the bud neck. Because we only captured images at a single timepoint, it is likely that these percentages are an underestimate of cells with Crz1 localized to the bud neck. These data suggest that Crz1 likely localizes to the bud neck prior to cell separation.

### 3.5. Crz1 Truncation Analysis Reveals Functionally Critical Regions

**Figure 5:**
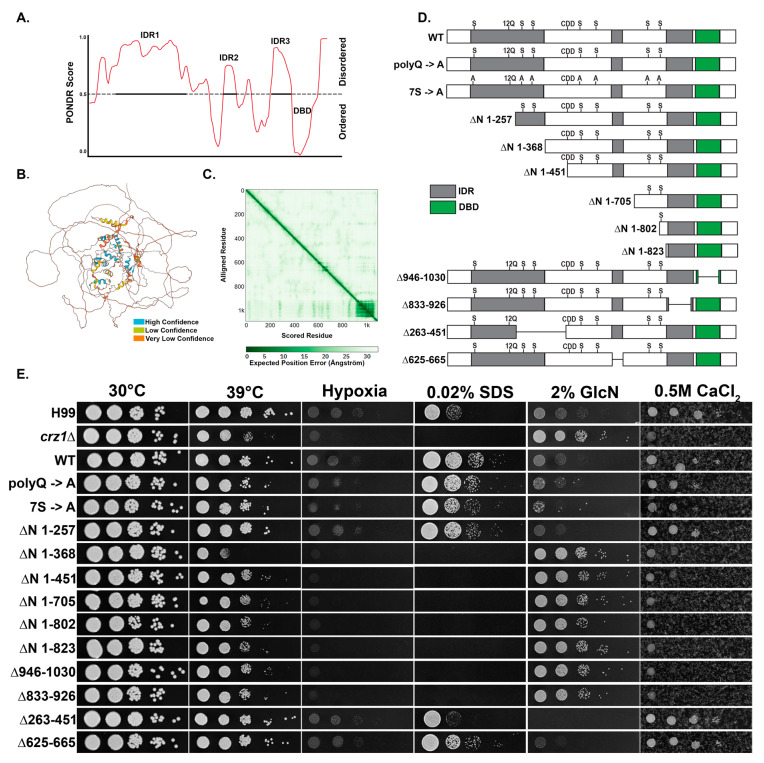
Mutational analysis of the Crz1 protein. (**A**) Results of PONDR (Predictor of Natural Disordered Regions) analysis of Crz1. A higher score correlates with higher disorder in the protein sequence. (**B**) AlphaFold prediction of Crz1 protein structure. The color pattern indicates the confidence level of the prediction. (**C**) The AlphaFold predicted aligned error plot shows dark green patches indicative of inter-protein interactions. (**D**) Protein diagrams of Crz1 mutant alleles used in this study. Green indicates the DNA binding domain (DBD) and grey indicates regions of intrinsic disorder (IDRs). The putative calcineurin-docking domain (CDD) [10], phosphorylation sites (S) [21], and polyQ region (12Q) are labeled. (**E**) The WT strain H99, the *crz1*Δ mutant, and mutant alleles in the *crz1*Δ mutant background were grown overnight in YPD, serially diluted, and spotted onto the media and incubated for two days as indicated.

The Crz1 protein contains a C-terminal DNA binding domain (DBD) which makes up less than 10% of the protein sequence, and several intrinsically disorder regions (IDRs) predicted by PONDR [24] (Figure 5A). Consistent with this domain prediction, AlphaFold software predicts one defined domain in the Crz1 protein sequence which corresponds to the DBD, and no other inter-protein interactions in the remaining sequence [25,26] (Figure 5B,C). The near N-terminal disordered sequence also contains a poly glutamine (polyQ) track which may contribute to granular localization [27,28]. In addition, seven serine phosphorylation sites which are known to be dephosphorylated by calcineurin add an additional layer of Crz1 regulation [21]. To identify what regions of the Crz1 protein sequence are important for its localization and function in response to different stresses, we created 10 internal (Δ) and N-terminal (ΔN) truncated Crz1 mutant alleles, as well as a polyQ mutant Crz1^(polyQ→A)^ (Figure 5D). We also utilized the existing phosphorylation site mutant Crz1^(7S→A)^ [21]. We then tagged these mutant proteins with mNeonGreen and introduced them into the *crz1*Δ mutant background to test for functional complementation. To minimize variations on gene expression caused by positional effects, we integrated all the constructs into the same “safe haven” *SH2* genetic locus [19,20]. For comparison, we also introduced the WT Crz1 protein sequence tagged with mNeonGreen into the *crz1*Δ mutant background using the same procedure. All Crz1 WT and mutant alleles were tested for (1) their ability to compensate for the loss of Crz1 in terms of growth in high temperatures, hypoxia, and media supplemented with calcium, Congo red, SDS, and glucosamine (Figure 5E) and (2) their subcellular localization in response to the various stresses (Table 1). For subcellular localization, a total of 100 cells were counted for each condition, and the percent of cells showing nuclear localization (as opposed to cytosolic), or granular localization are recorded in Table 1. Supplementary Figure S1 shows an example of cytosolic, nuclear, and granular localization of Crz1-mNeongreen.

As expected, the WT Crz1 construct was able to rescue all defects of the *crz1*Δ mutant examined (Figure 5E). To our surprise, mutations of the polyQ did not compromise functional complementation or granular localization. The Crz1^(polyQ→A)^ mutant protein showed reduced localization to the nucleus in response to 37˚C (32% vs. 99% in the nucleus, Table 1), but there was no discernable growth defect of this mutant strain even at 39 °C (Figure 5E). Similarly, mutation of the seven phosphorylation sites did not compromise complementation in any of the growth assays (Figure 5E). The Crz1^(7S→A)^ mutant expectedly displayed increased localization to the nucleus at 22°C (37% vs. 13% in WT) and after a 42 °C heat shock (100% of cells displayed nuclear localization vs. granular localization of the wildtype). In response to 1 M NaCl, 100% of cells showed Crz1^(7S→A)^ in granules, indicating these seven phosphorylation sites are not required for granular localization.

Deletions of the different intrinsically disordered regions had various impacts on the function and subcellular localization of Crz1. Interestingly, deletion of the first 257 amino acids, which includes part of IDR1 and the polyQ site, did not affect Crz1′s function in the conditions tested. However, the Crz1^(ΔN 1–257)^ mutant showed decreased granular localization in response to 42 °C heat shock (36% versus 100% in WT). Deletion of the second part of IDR1 (ΔN 263–451) had a negative effect on the ability of Crz1 to restore cell wall stress, but not other stresses (Figure 5E). Truncation of the first 368 amino acids (ΔN 1–368), which includes the entire IDR1 region, caused a total loss of function because the Crz1^(ΔN 1–368)^ mutant behaved exactly like the *crz1*Δ mutant in our spotting assays. The Crz1^(ΔN 1–368)^ protein was constitutively found in the nucleus, indicating that this region contains a possible nuclear exporting signal. Not surprisingly, the larger N-terminal truncation mutants, namely the Crz1^(ΔN 1–451)^ mutant, the Crz1^(ΔN 1–705)^ mutant, the Crz1^(ΔN 1–802)^ mutant, and the Crz1^(ΔN 1–823)^, mutant had the same phenotype as the *crz1*Δ mutant.

Deletion of the IDR2 region (Δ625–665) in the middle of the protein did not have any compromising effects in these growth assays, although its deletion abolished granular localization in response to 42 °C shock (0%) and slightly reduced granular localization in response to 1 M NaCl (84% vs. 99% in the WT). Strikingly, deletion of the IDR3 region (Δ833–926) caused constitutive nuclear localization as well as loss of function in spotting assays. Likewise, the DBD mutant, Crz1^(ΔN 946–1030)^, displayed similar localization and loss of function as the IDR3 region mutant Crz1^(ΔN 833–926)^. The IDR3 region is located directly upstream of the DBD and may play a critical role in its function. Notably, the IDR3 mutant allele Crz1^(ΔN 833–926)^, the DBD mutant allele, and the N-terminal truncated allele Crz1^(ΔN 1–368)^ were all not functional despite being constitutively localized to the nucleus, indicating these regions may all contribute to proper DNA binding and/or transcriptional activity.

## 4. Discussion

Lev et al. [8] first demonstrated that Crz1 localizes to granules in response to high salt or heat shock, and additionally, that Crz1 does not co-localize with polyA-binding protein Pab1 granules. Here, we found that Crz1 does co-localize with Pub1 granules. Our finding is consistent with previous findings in *S. cerevisiae* that Pab1 and Pub1 localize with different RNA binding proteins and/or stress granule components, indicating that the cytosol contains various types of RNP granules which may serve different functions [29,30].

Intrinsically disordered regions (IDRs) within proteins have been implicated as the drivers of granular assembly (or phase separation) [15,16]. For example, interactions between the three IDRs of the human stress granule assembly factor G3BP1 are regulated by phosphorylation, which will toggle the protein between closed and open states, the latter of which leads to stress granule assembly [15]. We identified three IDRs in the Crz1 protein sequence (Figure 5A), and individual Crz1 IDR truncations caused partial loss of stress granule localization (Figure 5B, Table 1). However, neither mutating the seven known phosphorylation sites (mimicking a dephosphorylated state) nor inhibiting the phosphatase calcineurin with FK506 (mimicking an enhanced phosphorylated state) prevented granular localization of Crz1, indicating a mechanism different from phosphorylation for regulation of phase separation. IDRs have been demonstrated to mediate transcription factor activity through liquid phase separation, DNA binding, or protein–protein interactions [13,14]. In our study, disruption of IDRs had deleterious effects on Crz1 function. For example, deletion of the IDR3 region located upstream of the DBD abolished Crz1 function in all assays (Figure 5B), indicating its critical role in mediating Crz1′s transcription factor activity.

Previously, it was known that Crz1 translocates to the nucleus in response to increasing temperature from room temperature to 37 °C. By examining cells expressing Crz1-mNeonGreen over time, we found that Crz1 may first localize to granules before nuclear translocation after the temperature shift (Figure 3A). Furthermore, Crz1 localizes to stress granules in addition to the nucleus in response to exogenous calcium at concentrations near host serum levels (Figure 3B). These data, together with our observations that Crz1 co-localizes with the phosphatase calcineurin to stress-induced granules, suggest that Crz1 localization to stress granules facilitates its translocation to the nucleus due to enhanced dephosphorylation by calcineurin. As multiple known targets of calcineurin localize to RNP granules [21], we suspect that granular localization of these proteins, including Crz1, may be a general mechanism to facilitate their dephosphorylation by calcineurin.

We also observed that Crz1 localizes to the bud neck under stress granule-inducing conditions, specifically in cells with larger buds (Figure 4). Interestingly, calcineurin also localizes to the bud neck and may play a critical role in coordinating cytokinesis and septation in *C. neoformans* under these stressful conditions through affecting its various substrate protein targets [22,31,32]. Like Crz1, another calcineurin substrate protein Cts1 co-localizes with calcineurin and Pub1 to cytoplasmic puncta and localizes to the bud neck of large budded cells [31,32]. Additionally, deletion of the *CTS1* gene caused defects in budding at 37 ˚C [32]. Calcineurin’s involvement in septation has also been demonstrated in *A. fumigatus* [33,34,35] and *Schizosaccharomyces pombe* [36]. Whether or not Crz1 plays an active role in septation or budding, either alone or in collaboration with other factors targeted by calcineurin, should be investigated in future studies.

Interestingly, loss of Crz1 does not confer any obvious growth defects in some of the conditions which induced granules (Figure 1). This is not surprising, as loss of Crz1 also does not clearly hamper cryptococcal growth at 37 °C or in the presence of 400 mM calcium [8] even though both conditions induce Crz1 nuclear translocation and activate its transcriptional activity [10]. While growth of the *crz1*Δ mutant does not appear to be sensitive at 37 °C, it is sensitive at 39 °C (Figure 5C). Moreover, double mutants of Crz1 and other calcineurin target proteins localized to granules caused thermosensitivity at 37 °C [21]. These observations suggest robust redundancy of calcineurin substrate protein function in ensuring cell separation and growth at higher temperatures.

## Figures and Tables

**Table 1:** Localization of Crz1 mutants.

	22 °C	37 °C	GlcN	NaCl		CaCl_2_	FK506		42 °C		42 °C + FK506	
**Strain**	N	N	N	N	G	N	N	G	N	G	N	G
**WT**	13%	99%	78%	1%	99%	97%	0%	5%	0%	100%	0%	100%
**7S -> A**	37%	100%	96%	0%	100%	100%	1%	0%	100%	0%	0%	100%
**polyQ -> A**	11%	32%	94%	0%	100%	98%	2%	2%	0%	100%	0%	92%
**∆N 1-257**	74%	95%	99%	10%	90%	100%	0%	4%	51%	36%	1%	57%
**∆N 1-368**	92%	100%	97%	100%	90%	97%	95%	0%	97%	0%	99%	0%
**∆N 1-451**	100%	100%	100%	15%	85%	98%	2%	0%	100%	0%	13%	87%
**∆N 1-705**	54%	87%	87%	100%	23%	100%	91%	0%	100%	0%	100%	0%
**∆N 1-802**	100%	100%	62%	100%	100%	98%	94%	0%	100%	0%	95%	0%
**∆N 1-823**	97%	100%	100%	97%	94%	100%	85%	0%	91%	0%	100%	0%
**∆NC 946-1030**	85%	94%	52%	100%	0%	98%	84%	0%	100%	0%	96%	0%
**∆NC 833-926**	73%	98%	100%	100%	100%	100%	100%	0%	100%	0%	100%	0%
**∆NC 625-665**	23%	82%	97%	5%	84%	85%	0%	23%	17%	0%	0%	20%
**% Different from WT**	0–20		>20		>40		>60		>80		100	

N: nuclear, G: Granular.

## Data Availability

Not applicable.

## Supplementary Materials

The following supporting information can be downloaded at: https://www.mdpi.com/article/10.3390/jof9020252/s1, Table S1: Strains, plasmids, and primers used; Figure S1: Crz1 cytosolic, nuclear, and granular localization Video S1: Crz1 response to 1 mM Calcium Video: Crz1 response to 1 mM Calcium; Video S3: Crz1 dynamic localization to the bud neck. Reference [37] is cited in the Supplementary Materials.

## References

[1] Thewes S. (2014). Calcineurin-Crz1 signaling in lower eukaryotes. Eukaryot. Cell.

[2] Choi J., Kim Y., Kim S., Park J., Lee Y.H. (2009). MoCRZ1, a gene encoding a calcineurin-responsive transcription factor, regulates fungal growth and pathogenicity of Magnaporthe oryzae. Fungal Genet. Biol..

[3] Cramer R.A., Perfect B.Z., Pinchai N., Park S., Perlin D.S., Asfaw Y.G., Heitman J., Perfect J.R., Steinbach W.J. (2008). Calcineurin target CrzA regulates conidial germination, hyphal growth, and pathogenesis of Aspergillus fumigatus. Eukaryot. Cell.

[4] Shwab E.K., Juvvadi P.R., Waitt G., Soderblom E.J., Barrington B.C., Asfaw Y.G., Moseley M.A., Steinbach W.J. (2019). Calcineurin-dependent dephosphorylation of the transcription factor CrzA at specific sites controls conidiation, stress tolerance, and virulence of Aspergillus fumigatus. Mol. Microbiol..

[5] Chen Y.L., Yu S.J., Huang H.Y., Chang Y.L., Lehman V.N., Silao F.G., Bigol U.G., Bungay A.A., Averette A., Heitman J. (2014). Calcineurin controls hyphal growth, virulence, and drug tolerance of Candida tropicalis. Eukaryot. Cell.

[6] Santos M., de Larrinoa I.F. (2005). Functional characterization of the Candida albicans CRZ1 gene encoding a calcineurin-regulated transcription factor. Curr. Genet..

[7] Moranova Z., Virtudazo E., Hricova K., Ohkusu M., Kawamoto S., Husickova V., Raclavsky V. (2014). The CRZ1/SP1-like gene links survival under limited aeration, cell integrity and biofilm formation in the pathogenic yeast Cryptococcus neoformans. Biomed. Pap. Med. Fac. Univ. Palacky Olomouc Czechoslov..

[8] Lev S., Desmarini D., Chayakulkeeree M., Sorrell T.C., Djordjevic J.T. (2012). The Crz1/Sp1 transcription factor of Cryptococcus neoformans is activated by calcineurin and regulates cell wall integrity. PLoS ONE.

[9] Adler A., Park Y.D., Larsen P., Nagarajan V., Wollenberg K., Qiu J., Myers T.G., Williamson P.R. (2011). A novel specificity protein 1 (SP1)-like gene regulating protein kinase C-1 (Pkc1)-dependent cell wall integrity and virulence factors in Cryptococcus neoformans. J. Biol. Chem..

[10] Chow E.W., Clancey S.A., Billmyre R.B., Averette A.F., Granek J.A., Mieczkowski P., Cardenas M.E., Heitman J. (2017). Elucidation of the calcineurin-Crz1 stress response transcriptional network in the human fungal pathogen Cryptococcus neoformans. PLoS Genet..

[11] Xu X., Lin J., Zhao Y., Kirkman E., So Y.-S., Bahn Y.-S., Lin X. (2017). Glucosamine stimulates pheromone-independent dimorphic transition in *Cryptococcus neoformans* by promoting Crz1 nuclear translocation. PLoS Genet..

[12] Stathopoulos-Gerontides A., Guo J.J., Cyert M.S. (1999). Yeast calcineurin regulates nuclear localization of the Crz1p transcription factor through dephosphorylation. Genes Dev..

[13] Brodsky S., Jana T., Barkai N. (2021). Order through disorder: The role of intrinsically disordered regions in transcription factor binding specificity. Curr. Opin. Struct. Biol..

[14] Brodsky S., Jana T., Mittelman K., Chapal M., Kumar D.K., Carmi M., Barkai N. (2020). Intrinsically Disordered Regions Direct Transcription Factor In Vivo Binding Specificity. Mol. Cell.

[15] Yang P., Mathieu C., Kolaitis R.-M., Zhang P., Messing J., Yurtsever U., Yang Z., Wu J., Li Y., Pan Q. (2020). G3BP1 Is a Tunable Switch that Triggers Phase Separation to Assemble Stress Granules. Cell.

[16] Protter D.S.W., Rao B.S., Van Treeck B., Lin Y., Mizoue L., Rosen M.K., Parker R. (2018). Intrinsically Disordered Regions Can Contribute Promiscuous Interactions to RNP Granule Assembly. Cell Rep..

[17] Zhang M., Zhu C., Duan Y., Liu T., Liu H., Su C., Lu Y. (2022). The intrinsically disordered region from PP2C phosphatases functions as a conserved CO_2_ sensor. Nat. Cell Biol..

[18] Fan Y., Lin X. (2018). Multiple Applications of a Transient CRISPR-Cas9 Coupled with Electroporation (TRACE) System in the *Cryptococcus neoformans* Species Complex. Genetics.

[19] Lin J., Fan Y., Lin X. (2020). Transformation of *Cryptococcus neoformans* by electroporation using a transient CRISPR-Cas9 expression (TRACE) system. Fungal Genet. Biol. FG B.

[20] Upadhya R., Lam W.C., Maybruck B.T., Donlin M.J., Chang A.L., Kayode S., Ormerod K.L., Fraser J.A., Doering T.L., Lodge J.K. (2017). A fluorogenic *C. neoformans* reporter strain with a robust expression of m-cherry expressed from a safe haven site in the genome. Fungal Genet. Biol. FG B.

[21] Park H.-S., Chow E.W.L., Fu C., Soderblom E.J., Moseley M.A., Heitman J., Cardenas M.E. (2016). Calcineurin Targets Involved in Stress Survival and Fungal Virulence. PLoS Pathog..

[22] Kozubowski L., Aboobakar E.F., Cardenas M.E., Heitman J. (2011). Calcineurin colocalizes with P-bodies and stress granules during thermal stress in *Cryptococcus neoformans*. Eukaryot. Cell.

[23] Kroschwald S., Munder M.C., Maharana S., Franzmann T.M., Richter D., Ruer M., Hyman A.A., Alberti S. (2018). Different Material States of Pub1 Condensates Define Distinct Modes of Stress Adaptation and Recovery. Cell Rep..

[24] Romero P., Obradovic Z., Li X., Garner E.C., Brown C.J., Dunker A.K. (2001). Sequence complexity of disordered protein. Proteins.

[25] Jumper J., Evans R., Pritzel A., Green T., Figurnov M., Ronneberger O., Tunyasuvunakool K., Bates R., Žídek A., Potapenko A. (2021). Highly accurate protein structure prediction with AlphaFold. Nature.

[26] Varadi M., Anyango S., Deshpande M., Nair S., Natassia C., Yordanova G., Yuan D., Stroe O., Wood G., Laydon A. (2022). AlphaFold Protein Structure Database: Massively expanding the structural coverage of protein-sequence space with high-accuracy models. Nucleic Acids Res..

[27] Lee C., Occhipinti P., Gladfelter A.S. (2015). PolyQ-dependent RNA-protein assemblies control symmetry breaking. J. Cell Biol..

[28] Marcelo A., Koppenol R., de Almeida L.P., Matos C.A., Nóbrega C. (2021). Stress granules, RNA-binding proteins and polyglutamine diseases: Too much aggregation?. Cell Death Dis..

[29] Buchan J.R., Muhlrad D., Parker R. (2008). P bodies promote stress granule assembly in *Saccharomyces cerevisiae*. J. Cell Biol..

[30] Mitchell S.F., Jain S., She M., Parker R. (2013). Global analysis of yeast mRNPs. Nat. Struct. Mol. Biol..

[31] Fox D.S., Cox G.M., Heitman J. (2003). Phospholipid-binding protein Cts1 controls septation and functions coordinately with calcineurin in *Cryptococcus neoformans*. Eukaryot. Cell.

[32] Aboobakar E.F., Wang X., Heitman J., Kozubowski L. (2011). The C2 domain protein Cts1 functions in the calcineurin signaling circuit during high-temperature stress responses in *Cryptococcus neoformans*. Eukaryot. Cell.

[33] Juvvadi P.R., Steinbach W.J. (2015). Calcineurin Orchestrates Hyphal Growth, Septation, Drug Resistance and Pathogenesis of *Aspergillus fumigatus*: Where Do We Go from Here?. Pathogens.

[34] Juvvadi P.R., Fortwendel J.R., Rogg L.E., Burns K.A., Randell S.H., Steinbach W.J. (2011). Localization and activity of the calcineurin catalytic and regulatory subunit complex at the septum is essential for hyphal elongation and proper septation in *Aspergillus fumigatus*. Mol. Microbiol..

[35] Juvvadi P.R., Fortwendel J.R., Pinchai N., Perfect B.Z., Heitman J., Steinbach W.J. (2008). Calcineurin localizes to the hyphal septum in *Aspergillus fumigatus*: Implications for septum formation and conidiophore development. Eukaryot. Cell.

[36] Lu Y., Sugiura R., Yada T., Cheng H., Sio S.O., Shuntoh H., Kuno T. (2002). Calcineurin is implicated in the regulation of the septation initiation network in fission yeast. Genes Cells Devoted Mol. Cell. Mech..

[37] Nielsen K., Cox G.M., Wang P., Toffaletti D.L., Perfect J.R., Heitman J. (2003). Sexual cycle of Cryptococcus neoformans var. grubii and virulence of congenic a and alpha isolates. Infect. Immun..

