# Supplementary material for: Molecular Dissection of Crz1 and Its Dynamic Subcellular Localization in Cryptococcus neoformans
Source: J Fungi (Basel). 2023 Feb 14;9(2):252. doi: 10.3390/jof9020252 (PMC9963361; doi:10.3390/jof9020252)
Supplement: Supplementary file 1 [file jof-09-00252-s001.zip › Supplemental Table S1.pdf]

Supplemental Table S1. Strains, plasmids, and primers used

***C. neoformans* strain name**	**Identifier**	**Reference**
H99α	H99α	(Nielsen et al., 2003)
crz1Δ	H99α, *CRZ1*::NAT^r	FGSC deletion set Plate 5 Well A7
BC402	H99α, *CRZ1*::NAT^r, P_{GPD1}-*CRZ1*-mNeonGreen-NEO^r	This Study
XX510	H99α, *CRZ1*::NAT^r, P_{GPD1}-mCherry-*CRZ1*-NEO^r	(Xu et al., 2017)
BC948	H99α, *CRZ1*::NAT^r, P_{GPD1}-*CRZ1*(7S -> A)-mNeonGreen-NEO^r	This Study
BC979	H99α, *CRZ1*::NAT^r, P_{GPD1}-*CRZ1*(polyQ -> A)-mNeonGreen-NEO^r	This Study
BC982	H99α, *CRZ1*::NAT^r, P_{GPD1}-*CRZ1*(ΔN 1-257)-mNeonGreen-NEO^r	This Study
BC1212	H99α, *CRZ1*::NAT^r, P_{GPD1}-*CRZ1*(ΔN 833-926)-mNeonGreen-NEO^r	This Study
BC1245	H99α, *CRZ1*::NAT^r, P_{GPD1}-*CRZ1*(ΔN 625-665)-mNeonGreen-NEO^r	This Study
BC1249	H99α, *CRZ1*::NAT^r, P_{GPD1}-*CRZ1*(ΔN 946-1030)-mNeonGreen-NEO^r	This Study
BC1252	H99α, *CRZ1*::NAT^r, P_{GPD1}-*CRZ1*(ΔN 1-802)-mNeonGreen-NEO^r	This Study
BC1256	H99α, *CRZ1*::NAT^r, P_{GPD1}-*CRZ1*(ΔN 1-451)-mNeonGreen-NEO^r	This Study
BC1258	H99α, *CRZ1*::NAT^r, P_{GPD1}-*CRZ1*(ΔN 1-705)-mNeonGreen-NEO^r	This Study
BC1259	H99α, *CRZ1*::NAT^r, P_{GPD1}-*CRZ1*(ΔN 1-823)-mNeonGreen-NEO^r	This Study
BC1273	H99α, *CRZ1*::NAT^r, P_{GPD1}-*CRZ1*(ΔN 1-368)-mNeonGreen-NEO^r	This Study
BC1310	H99α, *CRZ1*::NAT^r, P_{GPD1}-*CRZ1*(ΔN 263-451)-mNeonGreen-NEO^r	This Study
**Plasmid name**	**Identifier**	**Reference**
pXL1-mCherry-*CRZ1*	P_{GPD1}-mCherry-*CRZ1*-NEO^r	(Xu *et al.*, 2017)
pUC19-*CRZ1*-mNeonGreen	P_{GPD1}-*CRZ1*-mNeonGreen-NEO^r	This Study
LKB39	P_{H3}-GFP-*CNA1*-NAT^r	(Kozubowski et al., 2011)

LKB61	P_{GPD1}-mCherry-*CNA1-HYG^r^*	(Kozubowski *et al.*, 2011)
LKB88	P_{GPD1}-mCherry-*PUB1-HYG^r^*	(Kozubowski *et al.*, 2011)
**Primer name**	**Sequence (5'-> 3')**	**Purpose**
linlab3397/XX	taatggccggccatggcagatccagcctcacc	Cloning WT *CRZ1* open reading frame
linlab3398/XX	taatgcgatcgcatcctcttcactcgtttcactcttc	Cloning WT *CRZ1* open reading frame
Linlab7530/BC	agcggcagcggcagcggcagcggcagcggcagctggtggctgaccctcacttgc	Generate polyQ mutant
Linlab7953/BC	tgtcctggtgagtcaccctgtggtcaagaatgtgctatcaca	Generate DBD mutant
Linlab7954/BC	acagggtgactcaccaggaca	Generate DBD mutant
Linlab7761/BC	cacttctgtcgctacgctggtgcaagaactcggcgctg	Generate Δ833-926 mutant
Linlab7762/BC	cagcgtagcgacagaagtg	Generate Δ833-926 mutant
Linlab7765/BC	ataggccggccggcttccacagttaaccaaacg	Generate Δ1-257 mutant
Linlab7955/BC	ataggccggccatgaccaaccctaactctccc	Generate Δ1-368 mutant
Linlab7956/BC	ataggccggccatgggcaggggactatttgacgc	Generate Δ1-451 mutant
Linlab7959/BC	ataggccggccatggggttatatcctcatgcg	Generate Δ1-705 mutant
Linlab7957/BC	ataggccggccgctggtggttcaacgtgag	Generate Δ1-823 mutant
Linlab7961/BC	caatcaaatgggatggaggatatgatctgtatccagcccagt	Generate Δ263-451 mutant

Linlab7958/BC	atcctccatcccatttgattg	Generate Δ263-451 mutant
Linlab7962/BC	gttgagctcgccttctcc	Generate Δ625-665 mutant
Linlab7963/BC	ggagaaggcgagctcaacttgcatcagctcactcaatta	Generate Δ625-665 mutant

Kozubowski, L., Aboobakar, E.F., Cardenas, M.E., and Heitman, J. (2011). Calcineurin colocalizes with P-bodies and stress granules during thermal stress in *Cryptococcus neoformans*. Eukaryot Cell *10*, 1396-1402. 10.1128/ec.05087-11.

Nielsen, K., Cox, G.M., Wang, P., Toffaletti, D.L., Perfect, J.R., and Heitman, J. (2003). Sexual cycle of *Cryptococcus neoformans* var. *grubii* and virulence of congenic a and alpha isolates. Infection and immunity *71*, 4831-4841. 10.1128/iai.71.9.4831-4841.2003.

Xu, X., Lin, J., Zhao, Y., Kirkman, E., So, Y.-S., Bahn, Y.-S., and Lin, X. (2017). Glucosamine stimulates pheromone-independent dimorphic transition in *Cryptococcus neoformans* by promoting Crz1 nuclear translocation. PLOS Genetics *13*, e1006982. 10.1371/journal.pgen.1006982.
