# Supplementary material for: Molecular Dissection of Crz1 and Its Dynamic Subcellular Localization in Cryptococcus neoformans
Source: J Fungi (Basel). 2023 Feb 14;9(2):252. doi: 10.3390/jof9020252 (PMC9963361; doi:10.3390/jof9020252)
Supplement: Supplementary file 1 [file jof-09-00252-s001.zip › supplementary Figure S1.pdf]

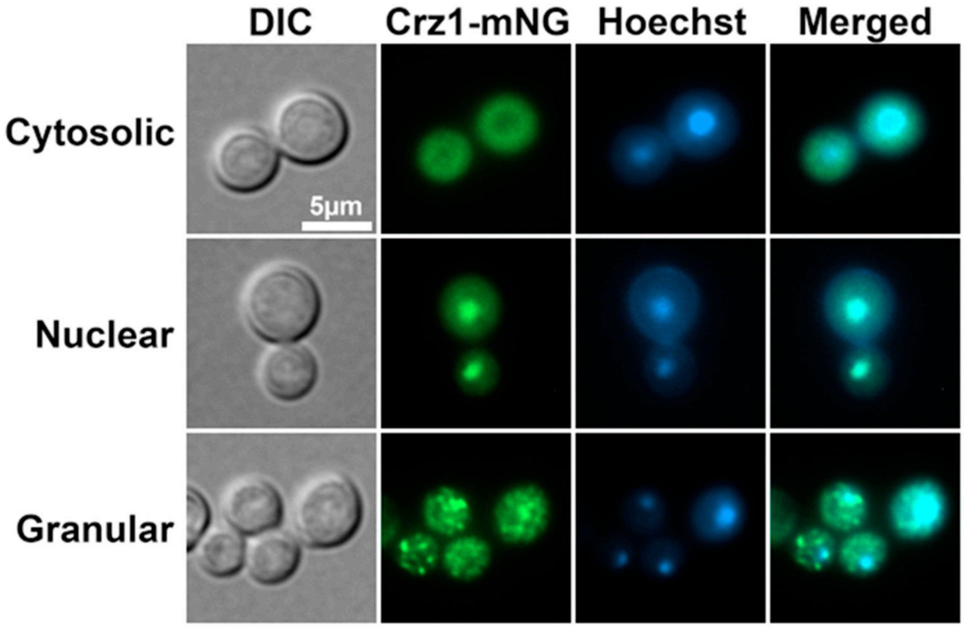

Supplementary Figure S1. Crz1 cytosolic, nuclear, and granular localization. Cells expressing Crz1-mNG were cultured overnight at 22°C in liquid YPD. To induce cytosolic localization of Crz1, FK506 was added to cells 30 minutes before preparing for microscopy, at a concentration of 1µg/ml. To induce nuclear or granular localization, the cells were washed and resuspended in water containing 100mM $CaCl_2$ or 1M NaCl, respectfully. To visualize the nucleus, Hoechst dye was added to a final concentration of 10µg/ml. After incubation in the dark for 15 minutes, cells were washed with water and prepared on slides for microscopy. The scale is the same for all images in the panel.
