# Supplementary figures and images for: Molecular Dissection of Crz1 and Its Dynamic Subcellular Localization in Cryptococcus neoformans
Source: J Fungi (Basel). 2023 Feb 14;9(2):252. doi: 10.3390/jof9020252 (PMC9963361; doi:10.3390/jof9020252)

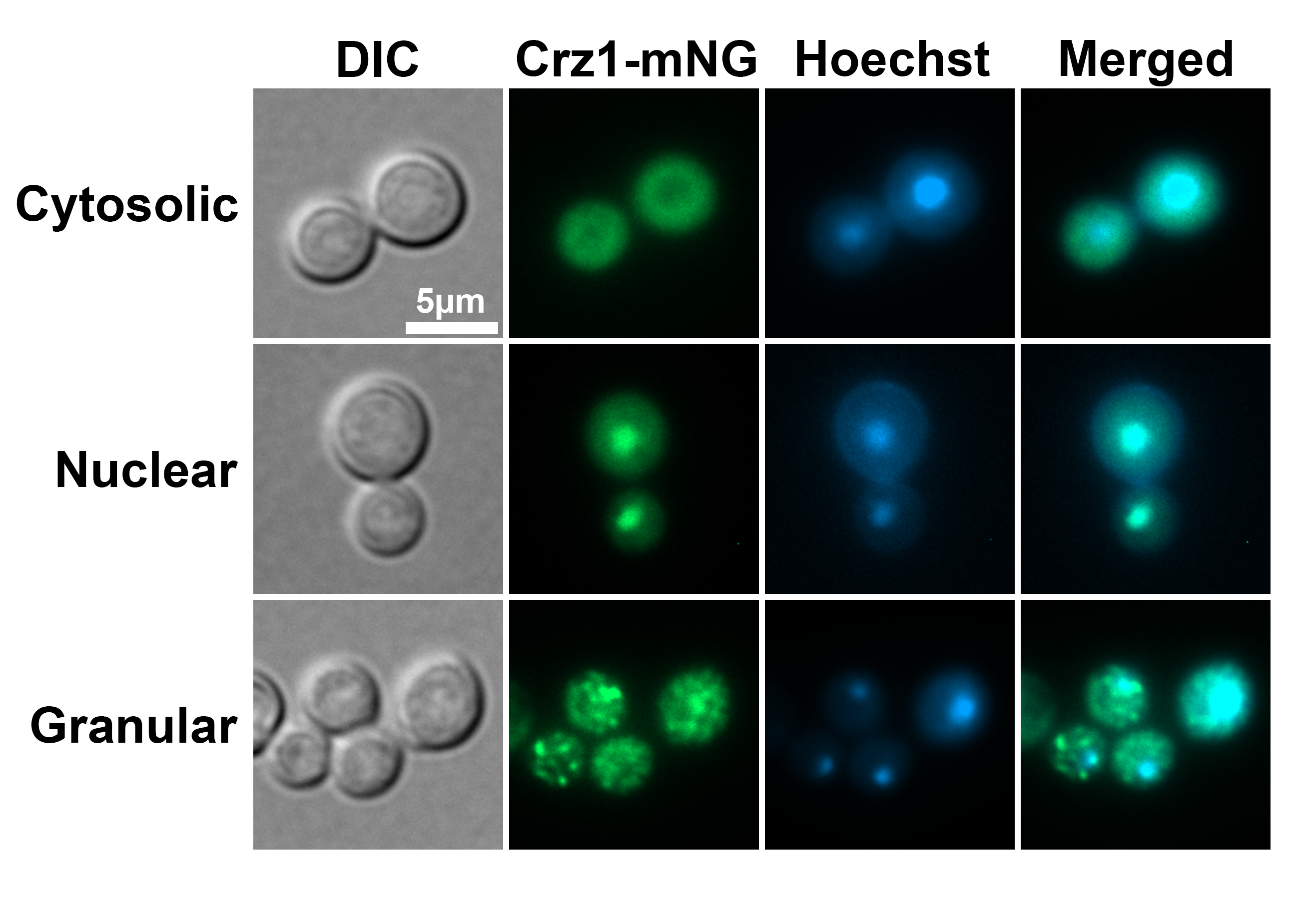

Supplement: Supplementary file 1 [file jof-09-00252-s001.zip › jof-2126216-supplementary revised/Supplemental_Figure_S1.png]
